# From the Lab to Real Life: Monitoring Cardiorespiratory Fitness during the COVID-19 Pandemic through Wearable Devices. An Exploratory Longitudinal Study on Healthy Participants

**DOI:** 10.3390/healthcare10040634

**Published:** 2022-03-28

**Authors:** Francesco Luciano, Valentina Cenacchi, Luca Ruggiero, Gaspare Pavei

**Affiliations:** 1 Department of Pathophysiology and Transplantation, University of Milan, 20133 Milan, Italy; luca.ruggiero@unimi.it (L.R.); gaspare.pavei@unimi.it (G.P.); 2 Faculty of Medicine, University of Milan, 20133 Milan, Italy; valentina.cenacchi@yahoo.it

**Keywords:** VO_2max_, VO_2peak_, containment stringency, SARS-CoV-2, epidemic, lockdown

## Abstract

COVID-19 containment measures hampered population cardiorespiratory fitness (which can be quantified as peak oxygen consumption ($\dot{V}$O_2peak_)) and the possibility to assess it using laboratory-based techniques. Although it is useful to ascertain the $\dot{V}$O_2peak_ recovery after lockdowns, the community and most scientific institutions were unable to evaluate it. Wearable devices may provide the opportunity to estimate cardiorespiratory fitness outside of the laboratory, without breaking self-isolation; herein, we explore the feasibility of this approach. Fifteen healthy participants were tested every 2 weeks for 10 weeks during a reduction of containment measures after a strict lockdown. Physical activity levels were measured using the International Physical Activity Questionnaire-Short Form (IPAQ-SF). $\dot{V}$O_2peak_ was estimated through a previously validated test based on the speed of a 60 m sprint run, the baseline-to-peak heart rate (HR) variation, and the velocity of HR decay after the sprint, and measured through a wearable HR monitor. Participants increased physical activity from the end of lockdown (1833 [917–2594] MET-min/week; median [1st quartile–3rd quartile]) until the end of follow-up (2730 [1325–3380] MET-min/week). The estimated $\dot{V}$O_2peak_ increased by 0.24 ± 0.19 mL/(min*kg*week) (regression coefficient ± standard error). Based on previous knowledge on the impact of inactivity on $\dot{V}$O_2peak_, our study indicates that a 10-week period of reducing the stringency of containment measures may not be sufficient to counteract the detrimental effects of the preceding lockdown.

## 1. Introduction

Epidemiology shows that physical activity reduces the risk of non-communicable diseases and premature mortality with a dose–response relation [1]. Physiology unveils the mechanisms underlying this association and the time they require to occur [2]. Both disciplines can benefit from the recent spread of mobile and wearable sensors, which allow the study of the impact of physical activity on populations and individuals. Smartphones and smartwatches equipped with GPS, accelerometers, pedometers, altimeters, and heart rate monitors provide constantly growing datasets on population-level physical activity [3]. Although they do not substitute laboratory studies in gold-standard conditions, wearable sensors favour research “from lab to real life” and vice versa, allowing everyday physiological functions to be measured, while actively encouraging a healthy lifestyle [4]. As such, they have become a precious source of information during the COVID-19 pandemic, which led many countries to implement containment measures in order to reduce the spread of the virus, but at the risk of impeding physical activity and increasing the risk of non-communicable diseases and overall mortality [2,5,6,7,8].

Physical inactivity has detrimental effects on many physiological systems; among others, it greatly curbs cardiorespiratory fitness, which can be measured as the maximal oxygen consumption during exercise ($\dot{V}$O_2max_) [2,9,10,11]. Considerable data on this topic come from extreme models of inactivity such as bed rest [2,10,11]. These experimental designs provide numerous advantages, such as being able to strictly control for confounding factors such as dietary requirements and countermeasures; however, they do not represent the nuanced real-life situations that occur during pandemic containment measures. Moreover, the direct measurement of $\dot{V}$O_2max_ is based on respirometry during incremental exercise tests, the use of which is limited in the present context. Indeed, this technique requires a laboratory, is time-demanding and implies a risk of SARS-CoV-2 transmission unless strict and costly hygiene protocols are employed [12]. In addition, the testing protocol is *per se* a strong training stimulus for most people, which makes it unsuitable for frequent and repeated testing when the goal is to assess the effect of reduced physical activity on $\dot{V}$O_2max_. In this regard, wearable devices could overcome the drawbacks of traditional experimental settings to assess cardiorespiratory fitness. If $\dot{V}$O_2max_ could be estimated with a short test that was accessible for most people without breaking self-isolation, cardiorespiratory fitness at the individual level could be easily and frequently monitored in real-life situations. This would be crucial to guide public health policies, countermeasures for inactivity, and the return to physical activity [13,14]. Storniolo and colleagues [15] developed a cardiorespiratory function test based on the heart rate (HR) off-kinetics measured immediately after a maximal 60 m sprint run using a low-cost wearable device that continuously recorded the HR. The test was validated against incremental $\dot{V}$O_2max_ running tests performed on a treadmill [15] and satisfied all of the above-mentioned criteria: it was short, it could be performed autonomously by the participants, and it used low-cost and widely available wearable devices.

The aims of this investigation were (i) to evaluate the feasibility of a 60 m sprint test in an exploratory longitudinal study conducted immediately after a lockdown, maintaining participants’ self-isolation and (ii) to quantify how reducing the stringency of the containment impacted on cardiorespiratory fitness through time. We hypothesised that the progressive lowering of the stringency of containment measures would lead to increased physical activity and higher cardiorespiratory fitness; the available literature did not provide information regarding the expected magnitude of this effect.

## 2. Materials and Methods

### 2.1. Study Design and Participants

**Figure 1:**
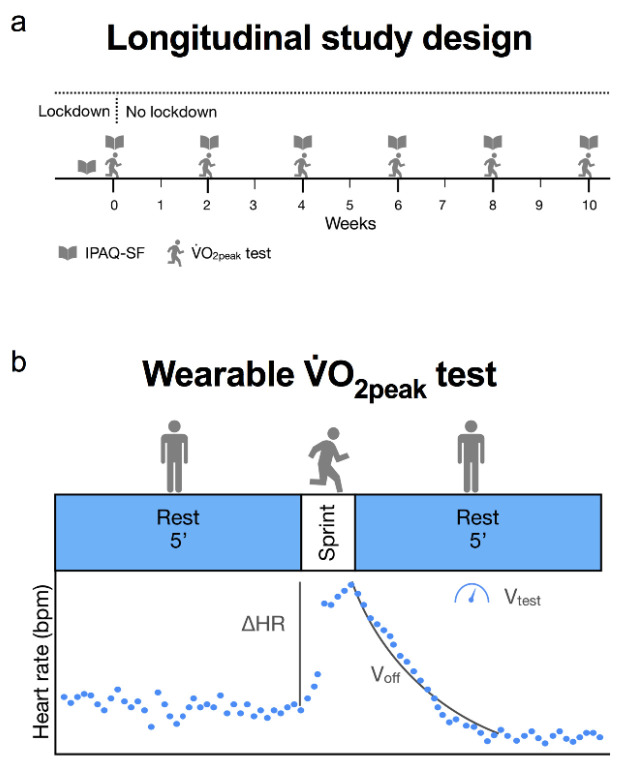
(**a**) Exploratory longitudinal study design. Every two weeks, physical activity and cardiorespiratory fitness ($\dot{V}$O_2peak_) were assessed through the International Physical Activity Level Questionnaire-Short Form (IPAQ-SF) and the 60 m sprint test, respectively. (**b**) Schematic representation of the protocol used to estimate $\dot{V}$O_2peak_ [15]. A wearable device recorded the HR before, during, and after a 60-metre sprint run. The baseline-to-peak HR difference (ΔHR), the velocity of HR decay after the sprint (v_off_), and the mean 60 m sprint speed (v_test_) were used as predictors.

An exploratory longitudinal study design was conducted on a cohort of fifteen healthy participants (4 females, 11 males; 24 ± 4 years, mean ± SD). The study was held in Italy after 2 months of strict lockdown. Participants were recruited in May 2020 and observed for 10 weeks, during a reduction of COVID-19 containment measures, until the end of July 2020 (Figure 1a). Every two weeks, physical activity was evaluated through a smartphone-delivered International Physical Activity Questionnaire-Short Form (IPAQ-SF). The decision to use this questionnaire was based on its well-documented reliability and validity [16,17]. The wearable devices could have been used to estimate physical activity, but their results would have been less interpretable due to the use of proprietary algorithms and the fact that participants may not have worn them during the entire day. This risk was assumed as especially high during home-confinement periods, during which IPAQ-SF was deemed more reliable. In addition, participants also performed a 60 m running sprint test to estimate peak oxygen consumption ($\dot{V}$O_2peak_) [15].

Healthy, physically active participants aged between 18 and 40 years were considered eligible. Participants were excluded if they had had COVID-19 at any moment during the previous lockdown, if they had any disease or clinical condition that was incompatible with sprinting and $\dot{V}$O_2peak_ testing, if their body mass index was above 30 kg/m^2^, or if they were living outside Italy during the study period. All the participants gave written informed consent after becoming aware of the potential risks involved in the experimental sessions. In order to address a potential source of bias, each participant performed all of the $\dot{V}$O_2peak_ tests at the same hour of the day on the same straight, flat asphalt track. The environmental temperature was expected to rise during the follow-up period as this study was conducted from May to August in the northern hemisphere. Despite this, the decision to perform all of the tests at a fixed hour of the day was made in order to minimise the possible confounding impacts of circadian variations on the variables analysed to assess cardiorespiratory fitness. The reporting of this research study was also performed in compliance with the STROBE checklist [18], which is included in the Supplementary Materials.

### 2.2. Measurement Instruments

Throughout the study period, the varying stringency of the COVID-19 containment measures was quantified by using the publicly available data on the stringency index [18]. This composite measure is based on many indicators including school closures, workplace closures, cancelled public events, restrictions on gatherings, closed public transport, public information campaigns, stay-at-home recommendations or requirements, restrictions on internal movement, and face coverings. It ranges from 0 (minimal stringency) to 100 (maximal stringency) [19].

Physical activity was evaluated using the IPAQ-SF, which referred to the previous 14 days and assessed walking, moderate-intensity activities, vigorous-intensity activities, and sedentary behaviour. Data were analysed according to the IPAQ scoring guidelines [20]. Physical activity was estimated by multiplying the total amount of minutes spent per week by the metabolic equivalent score of each activity (MET) and expressed as MET-min/week.

Cardiorespiratory fitness was estimated as $\dot{V}$O_2peak_ through the method published by Storniolo and colleagues [15]. Briefly, the participants performed a 5 min resting measurement in a standing position followed by a maximal 60 m sprint run and an additional 5 min resting measurement; a personal wearable device (Garmin Fenix 5 plus, Garmin Edge 305 or Polar m430) continuously recorded the HR every second during the rest and test periods (Figure 1b). Participants were familiarised with the test protocol in a separate session prior to the first acquisition; then, they performed the sprint task autonomously and the data were analysed remotely. The data were visually reviewed and if gaps in the HR data were detected, participants were asked to repeat the measurement. In the case of any errors, the participants had to wait 24 h before repeating the sprint test. $\dot{V}$O_2peak_ was then estimated as:

(1) $$\dot{V}O_{2_{\mathrm{peak}}}=7.46*v_{\mathrm{test}}+261.4*v_{\mathrm{off}}-0.19*\Delta \mathrm{HR}$$

where v_test_ (m s^−1^) is the mean velocity over 60 metres calculated using the built-in stopwatch feature, v_off_ (s^−1^) is the velocity of heart rate (HR) decay after the sprint, and ΔHR is the difference between the HR at the start of the sprint and at the beginning of the off-kinetics (Figure 1b). v_off_ was calculated from an exponential fit of the HR off-kinetics after maximal sprint as:

(2) $$v_{\mathrm{off}}=\frac{1}{\tau }$$

where *τ* is the time from the end of the sprint to reach 37% of the maximum excursion of the HR. The analysis of the HR kinetics was performed with R 3.6.2 and R Studio [21,22].

### 2.3. Statistical Analysis

A linear mixed model was implemented in order to assess the variation of estimated $\dot{V}$O_2peak_ during time in a within-subject design, by controlling for IPAQ level. Assumptions for this model were checked and reported in the model diagnostics plots as Supplementary Figure S1. The regression analysis was performed with R 3.6.2 and R Studio [21,22], using the libraries lme4 for the mixed-effect model and DHARMa for the diagnostic plots [23,24]. As indicated in lme4 documentation and due to the limited interpretability of *p*-values, especially in exploratory observational studies [23,25], results of the regression model were reported as regression coefficients, standard errors, and t-statistics in addition to their *p*-value.

## 3. Results

**Figure 2:**
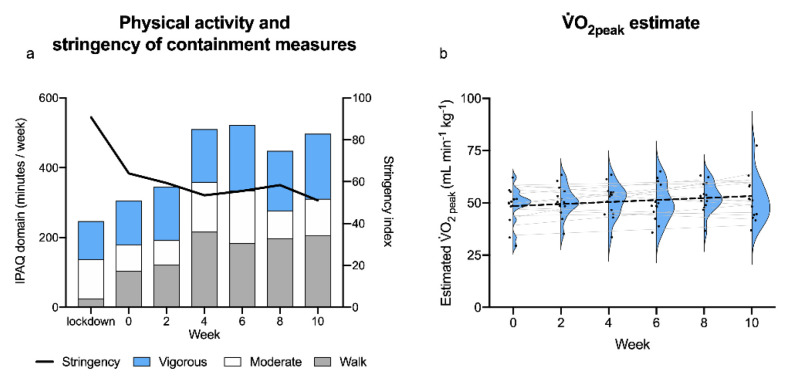
(**a**) Physical activity and stringency of containment measures. During the study period, the stringency of the COVID-19 containment measures fell (black line), while participants increased their total physical activity levels (bars: average time doing walking, moderate, or vigorous activity). (**b**) Estimated $\dot{V}$O_2peak_ throughout the follow-up. The estimated $\dot{V}$O_2peak_ increased by ∼0.7%/week (t-statistic: 1.3); individual data points, individual and mean regression lines (thin and thick, respectively), and probability density functions (violin contours) are reported.

One participant completed only three $\dot{V}$O_2peak_ tests, so her data were excluded from analysis. The remaining participants completely concluded their follow-up, taking part in six $\dot{V}$O_2peak_ tests and six IPAQ-SF measurements each. The demographic and anthropometric characteristics of the 14 analysed participants are summarised in Supplementary Table S1 [18]. During the 10 weeks of follow-up, containment measures became progressively less stringent (Figure 2a). The participants reduced their sitting time and increased their physical activity from end of lockdown (10.5 [10,11,12] hours/day sitting and 1833 [917–2594] MET-min/week of physical activity; median [1^st^ quartile–3^rd^ quartile]) until the end of follow-up (8 [7,8,9,10,11] hours/day sitting and 2730 [1325–3380] MET-min/week). This increase was mainly due to greater time spent walking, with smaller variations in moderate and vigorous physical activity (Figure 2a). The estimated $\dot{V}$O_2peak_ increased by 0.24 ± 0.19 mL/(kg*min) (regression coefficient ± standard error; t-statistic: 1.3; *p*-value: 0.21) per week, with a weekly average increase of 0.7% from the values recorded immediately post-lockdown. Figure 2b shows the individual and mean variations in cardiorespiratory fitness during the follow-up period and the extended summary statistics for the outcome variables are provided in Supplementary Tables S2–S4.

## 4. Discussion

In this 10-week longitudinal study, fifteen healthy participants performed a test to estimate cardiorespiratory fitness through a wearable HR device. Since the measurements from one participant were excluded, only fourteen subjects were included in the analysis. When resuming physical activity after a lockdown, their estimated $\dot{V}$O_2peak_ increased by 0.24 ± 0.19 mL/(kg*min) per week, or 0.7% per week from the values recorded immediately post-lockdown.

Containment measures can make people lose their cardiorespiratory fitness as a consequence of physical inactivity; no data are available about how fast $\dot{V}$O_2peak_ is lost in this condition, but an estimate can be drawn from some experimental models of physical inactivity. When under forced bed rest, people can lose $\dot{V}$O_2peak_ by up to ∼3.5% per week [2,10,11]; when daily step numbers are reduced to 1500 per day, $\dot{V}$O_2peak_ decreases by ∼2.5% per week [26,27], hinting that a minimum dose of activity is required to maintain cardiorespiratory fitness. In the present investigation, participants increased their $\dot{V}$O_2peak_ by ∼0.7% per week indicating that simply returning to an active lifestyle may not be sufficient to counteract the detrimental effect of previous containment measures. The observed increment of 0.24 ± 0.19 mL/(kg*min) per week is lower than the increments up to ∼0.4 mL/(kg*min) per week reported with high-intensity programmes [28]—possibly because the latter were structured exercise interventions—but still higher than observed when structured exercise interventions were unsupervised [29]. The participants of this study already performed on average ∼200 min of moderate and vigorous physical activity during lockdown (a similar amount to that suggested by the World Health Organization guidelines for physical activity [30]) and mainly incremented their walking time during the study period.

The sample size is the main limitation affecting the findings presented above. However, the results from this exploratory longitudinal study can be used to plan research with larger cohorts assessing the resumption of physical activity and cardiorespiratory fitness after a pandemic—or any prolonged period of inactivity—using wearable devices. The observed effect is small in this exploratory study; studies with larger cohorts and gold-standard examinations are needed to further test the findings in this study. Moreover, this study focused only on young healthy participants. In order to increase the generalizability of results, further investigations could also include other population groups.

Not only did the current pandemic impact on physical activity, but the use of traditional laboratory-based methods to gather information on these impacts was also hampered. Direct $\dot{V}$O_2max_ quantification presents considerable limitations when the aim is to assess cardiorespiratory fitness between intermittent lockdowns or periods of inactivity. Estimating cardiorespiratory fitness through wearable devices can fill the gap between wide-scale epidemiological evidence and laboratory-based physiological experiments, as a low-cost and COVID-safe alternative. This can be combined with the population-level tracking of physical activity to examine trends in cardiorespiratory fitness, tailor the stringency of containment measures and guide the return to physical activity for patients, athletes, and the general population.

## Data Availability

The data presented in this study are available from the corresponding author upon request.

## Supplementary Materials

The following are available online at https://www.mdpi.com/article/10.3390/healthcare10040634/s1, Figure S1: Model diagnostics for the mixed linear model, Table S1: Demographic and anthropometric characteristics of the participants, Table S2: Descriptive statistics for physical activity, Table S3: Descriptive statistics for sedentary behaviour, Table S4: Descriptive statistics for $\dot{V}$O_2peak_, STROBE (STrengthening the Reporting of OBservational studies in Epidemiology) checklist.
