# Supplementary material for: From the Lab to Real Life: Monitoring Cardiorespiratory Fitness during the COVID-19 Pandemic through Wearable Devices. An Exploratory Longitudinal Study on Healthy Participants
Source: Healthcare (Basel). 2022 Mar 28;10(4):634. doi: 10.3390/healthcare10040634 (PMC9028944; doi:10.3390/healthcare10040634)
Supplement: Supplementary file 1 [file healthcare-10-00634-s001.zip › healthcare-1642358-supplementary.pdf]

SUPPLEMENTARY MATERIAL

Materials and methods

Statistical analysis

A linear mixed model was implemented in order to assess the variation of estimated $\dot{V}O_{2peak}$ during time in a within-subject design, by controlling for IPAQ level. Supplementary Figure S1 reports the inherent model diagnostics plots.

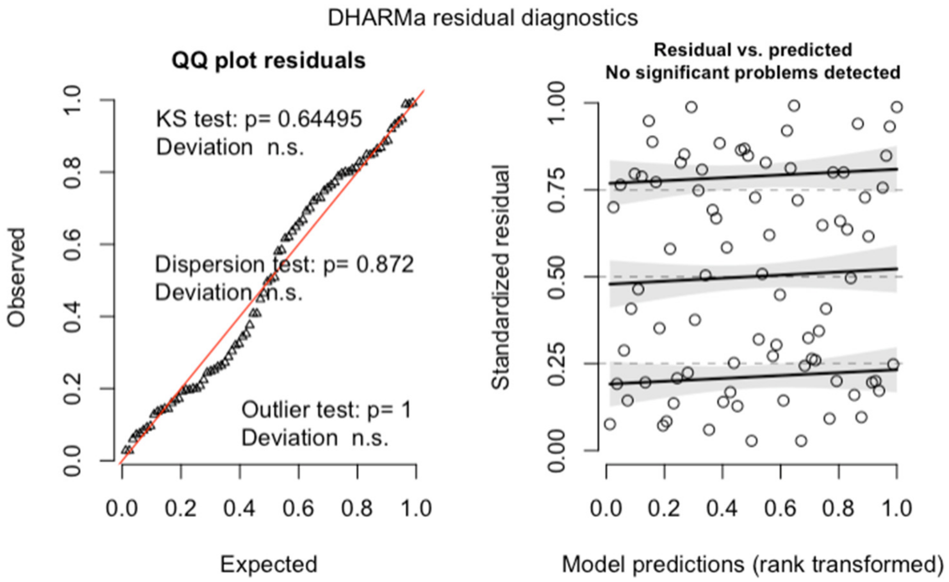

Supplementary Figure S1. Model diagnostics for the mixed linear model regressing $\dot{V}O_{2peak}$ over test week while controlling for IPAQ score, with within-subjects as a random factor. Library: "DHARMa" for R.

Results

The demographic and anthropometric characteristics of the 14 analysed participants are reported in Supplementary Table S1.

	Age (yrs)	*Body height (m)*	*Body mass (kg)*
1st quartile	20.5	1.71	60.0
Median	23.0	1.76	63.0
3rd quartile	24.8	1.78	73.5
Mean	23.8	1.74	66.4
Standard deviation	4.3	0.06	8.1

Supplementary Table S1. Demographic and anthropometric characteristics of the 14 analysed participants.

In addition to the results mentioned in the main text, extended summary statistics for the outcome variables are provided in Supplementary Tables S2, S3, and S4.

	Lockdown	*Follow-up week 0*	*Follow-up week 2*	*Follow-up week 4*	*Follow-up week 6*	*Follow-up week 8*	*Follow-up week 10*
1st quartile	917	1301	1291	1568	1868	1276	1325
Median	1833	2113	1937	2232	2306	2316	2730
3rd quartile	2594	2443	3088	3190	2897	2887	3380
Mean	1767	1952	2244	2511	3067	2973	3126
Standard deviation	1151	1151	1380	1230	2592	2416	2469

Supplementary Table S2. Descriptive statistics for physical activity, measured as metabolic equivalent of task per week (MET-min/week).

	Lockdown	*Follow-up week 0*	*Follow-up week 2*	*Follow-up week 4*	*Follow-up week 6*	*Follow-up week 8*	*Follow-up week 10*
1st quartile	10.0	8.3	9.0	8.0	9.0	8.3	7.0
Median	10.5	10.0	10.0	9.5	10.0	9.5	8.0
3rd quartile	12.0	10.8	10.8	10.0	12.0	11.0	10.8
Mean	11.2	9.6	9.9	9.5	9.9	9.5	9.1
Standard deviation	3.3	2.0	2.2	2.3	2.9	2.8	3.3

Supplementary Table S3. Descriptive statistics for sedentary behaviour, measured as average hours spent sitting per day.

	Follow-up week 0	*Follow-up week 2*	*Follow-up week 4*	*Follow-up week 6*	*Follow-up week 8*	*Follow-up week 10*
1st quartile	48	48	45	46	49	44
Median	50	50	53	48	53	51
3rd quartile	52	55	55	59	58	58
Mean	49	51	51	50	53	51
Standard deviation	9	7	8	9	7	11

Supplementary Table S4. Descriptive statistics for $\dot{V}O_{2peak}$, measured as estimated mLO_2 kg^{-1} min^{-1}, for each week.

STROBE (STrengthening the Reporting of OBservational studies in Epidemiology) checklist

STROBE Statement—Checklist of items that should be included in reports of ***cohort studies***

	Item No	Recommendation	Page No
**Title and abstract**	1	(*a*) Indicate the study's design with a commonly used term in the title or the abstract	1
		(*b*) Provide in the abstract an informative and balanced summary of what was done and what was found	1
**Introduction**			
Background/rationale	2	Explain the scientific background and rationale for the investigation being reported	1-2
Objectives	3	State specific objectives, including any prespecified hypotheses	2
**Methods**			
Study design	4	Present key elements of study design early in the paper	3
Setting	5	Describe the setting, locations, and relevant dates, including periods of recruitment, exposure, follow-up, and data collection	2-4
Participants	6	(*a*) Give the eligibility criteria, and the sources and methods of selection of participants. Describe methods of follow-up	2-3
		(*b*) For matched studies, give matching criteria and number of exposed and unexposed	
Variables	7	Clearly define all outcomes, exposures, predictors, potential confounders, and effect modifiers. Give diagnostic criteria, if applicable	2-4
Data sources/ measurement	8*	For each variable of interest, give sources of data and details of methods of assessment (measurement). Describe comparability of assessment methods if there is more than one group	3-4

Bias	9	Describe any efforts to address potential sources of bias	3-4
Study size	10	Explain how the study size was arrived at	2-3
Quantitative variables	11	Explain how quantitative variables were handled in the analyses. If applicable, describe which groupings were chosen and why	3-4
Statistical methods	12	(*a*) Describe all statistical methods, including those used to control for confounding<br>(*b*) Describe any methods used to examine subgroups and interactions<br>(*c*) Explain how missing data were addressed<br>(*d*) If applicable, explain how loss to follow-up was addressed<br>(*e*) Describe any sensitivity analyses	4
**Results**			
Participants	13*	(a) Report numbers of individuals at each stage of study—eg numbers potentially eligible, examined for eligibility, confirmed eligible, included in the study, completing follow-up, and analysed<br>(b) Give reasons for non-participation at each stage<br>(c) Consider use of a flow diagram	4
Descriptive data	14*	(a) Give characteristics of study participants (eg demographic, clinical, social) and information on exposures and potential confounders<br>(b) Indicate number of participants with missing data for each variable of interest<br>(c) Summarise follow-up time (eg, average and total amount)	4; Supplementary Materials
Outcome data	15*	Report numbers of outcome events or summary measures over time	4-5; Supplementary Materials

Main results	16	(*a*) Give unadjusted estimates and, if applicable, confounder-adjusted estimates and their precision (eg, 95% confidence interval). Make clear which confounders were adjusted for and why they were included<br>(*b*) Report category boundaries when continuous variables were categorized<br>(*c*) If relevant, consider translating estimates of relative risk into absolute risk for a meaningful time period	4-5; Supplementary Materials
Other analyses	17	Report other analyses done—eg analyses of subgroups and interactions, and sensitivity analyses	
**Discussion**			
Key results	18	Summarise key results with reference to study objectives	5
Limitations	19	Discuss limitations of the study, taking into account sources of potential bias or imprecision. Discuss both direction and magnitude of any potential bias	5-6
Interpretation	20	Give a cautious overall interpretation of results considering objectives, limitations, multiplicity of analyses, results from similar studies, and other relevant evidence	5-6
Generalisability	21	Discuss the generalisability (external validity) of the study results	6
**Other information**			
Funding	22	Give the source of funding and the role of the funders for the present study and, if applicable, for the original study on which the present article is based	5

*Give information separately for exposed and unexposed groups.

Note: An Explanation and Elaboration article discusses each checklist item and gives methodological background and published examples of transparent reporting. The STROBE checklist is best used in conjunction with this article (freely available on the Web sites of PLoS Medicine at http://www.plosmedicine.org/, Annals of Internal Medicine at http://www.annals.org/, and Epidemiology at http://www.epidem.com/). Information on the STROBE Initiative is available at http://www.strobe-statement.org.
